# Designing the Self‐Assembly of Disordered Materials Via Color Frustration

**DOI:** 10.1002/adma.202502136

**Published:** 2025-06-10

**Authors:** Andreas Neophytou, Francesco Sciortino, John Russo

**Affiliations:** ^1^ Dipartimento di Fisica Sapienza Università di Roma Piazzale Aldo Moro 5 Roma 00185 Italy

**Keywords:** amorphous materials, glass, material design, programmed self‐assembly, patchy particles, phase transition

## Abstract

The ability to control self‐assembly with atomic‐level precision has led to remarkable advances in the rational design of crystalline materials. However, similar design strategies have yet to be developed for amorphous materials. Here, a strategy is devised for programming the self‐assembly of amorphous structures by encoding frustration into the building block design. The building blocks are tailored to locally favor the formation of five‐member rings and yield a network of face‐sharing dodecahedral cages whose icosahedral symmetry prevents long‐range order. Surprisingly, unlike geometrically frustrated glasses that form through kinetic arrest, the network nucleates spontaneously from the liquid phase, representing a novel type of thermodynamically stable disordered phase. The stabilization of this frustrated phase via programmable interactions paves the way for a new generation of disordered materials.

## Graphical abstract

What if disorder can be designed? This work introduces a strategy to self‐assemble amorphous materials by designing bonding rules for patchy particles that suppress crystallinity and promote frustration. The designer particles nucleate a network of dodecahedral cages from the liquid, yielding a novel equilibrium amorphous phase. This approach redefines how disorder and function can be programmed at the colloidal scale.

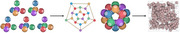

## 1 Introduction

Recent advances in understanding and controlling self‐assembly processes have opened up the possibility of rationally designing building blocks – whether they be organic molecules, proteins or nanoparticles – to reliably organize into a target structure. By encoding “assembly information” into the building blocks, it is possible to precisely control their spatial organization, which can reach atomic‐scale resolution.^[^ ^1^, ^2^ ^]^ From DNA‐based nanostructures,^[^ ^3^, ^4^ ^]^ to protein superlattices,^[^ ^2^ ^]^ to colloidal crystals,^[^ ^5^ ^]^ there has been huge success in programming the self‐assembly of ordered structures that are finite^[^ ^6^, ^7^, ^8^ ^]^ and infinite^[^ ^5^, ^9^, ^10^, ^11^ ^]^ in size. However, the prospect of rationally designing disordered structures with the same kind of precision remains an exciting new frontier.^[^ ^12^, ^13^, ^14^ ^]^ Developing self‐assembly strategies for amorphous materials could present new possibilities in areas like structural color,^[^ ^15^, ^16^ ^]^ mechanical metamaterials,^[^ ^17^, ^18^ ^]^ and phase change materials.^[^ ^19^, ^20^ ^]^

Building block designs for crystalline materials favor the formation of structural motifs related to the desired crystal's unit cell while disfavoring those of competing structures.^[^ ^11^, ^21^, ^22^, ^23^, ^24^, ^25^ ^]^ Establishing a design for amorphous materials is not as straightforward since they do not have periodically repeating structural motifs to target. Previous work has looked to control the short‐range order in amorphous structures by tuning the building block's valency and shape.^[^ ^26^, ^27^, ^28^, ^29^ ^]^ However, structures with identical short‐range order can exhibit different material properties,^[^ ^30^, ^31^ ^]^ meaning building block designs for amorphous materials should encode ordering that goes beyond the short‐range, such as bond orientational order,^[^ ^32^, ^33^, ^34^ ^]^ hyperuniformity^[^ ^35^, ^36^ ^]^ or medium‐range order.^[^ ^37^, ^38^ ^]^ In the context of programming self‐assembly, medium‐range order is of particular interest. It arises when locally favored structural units cluster to produce extended regions of organization.^[^ ^37^, ^38^, ^39^, ^40^, ^41^, ^42^ ^]^ These structural units usually have pentagonal or icosahedral symmetry meaning they cannot tile 3D Euclidean space (unlike the unit cell of a crystal) giving rise to geometric frustration and preventing the development of long‐range order.^[^ ^43^, ^44^, ^45^, ^46^, ^47^ ^]^ However, frustration can be avoided if these non‐space‐filling units are combined with certain space‐filling structural motifs, as is the case in clathrate crystals,^[^ ^48^, ^49^ ^]^ Frank‐Kasper phases^[^ ^50^, ^51^, ^52^, ^53^ ^]^ and quasicrystals.^[^ ^10^, ^54^ ^]^ Therefore, it should be possible to rationally design amorphous materials with specific medium‐range order by promoting the formation of non‐space‐filling structural units while suppressing space‐filling ones, thereby encoding geometric frustration into the building blocks.

To be programmable building blocks must have tunable interactions which can be precisely tailored to favor the formation of a target structure. One such class of building blocks are colloidal patchy particles, nano‐ to micron‐scale particles that have a repulsive core decorated with attractive sites (or “patches”) which mediate inter‐particle bonding.^[^ ^26^, ^34^, ^55^ ^]^ The versatility of patchy particles stems from the number of orthogonal design handles they possess, such as the geometry of the repulsive core, the relative position of the patches, the geometry of the patches or the strength of the patch‐patch interactions.^[^ ^27^, ^34^, ^56^ ^]^ Beyond colloids, they serve as coarse‐grained proxies for protein‐protein and nucleic‐acid interactions^[^ ^57^, ^58^, ^59^ ^]^ and underpin innovative strategies for materials design and programmed self–assembly.^[^ ^5^, ^60^, ^61^, ^62^ ^]^ Recent developments in DNA nanotechnology^[^ ^3^, ^4^ ^]^ have lead to the synthesis of DNA‐based patchy particles^[^ ^11^, ^63^, ^64^ ^]^ made from polyhedral DNA‐origami whose vertices are functionalized with single‐stranded DNA (ssDNA) overhangs to act as the attractive patches. Utilizing DNA as the synthetic ingredient for patchy particles offers the possibility of “coloring” the particles and their patches, where different colors are used to represent particles and patches of different DNA sequences. Since interacting patches must have complementary sequences, through such coloring it is possible to introduce specific interactions into a patchy particle system. Current state‐of‐the‐art in DNA‐nanotechnology allows the synthesis of mixtures of patchy particles with many components.^[^ ^11^ ^]^

## 2 Results

**Figure 1:**
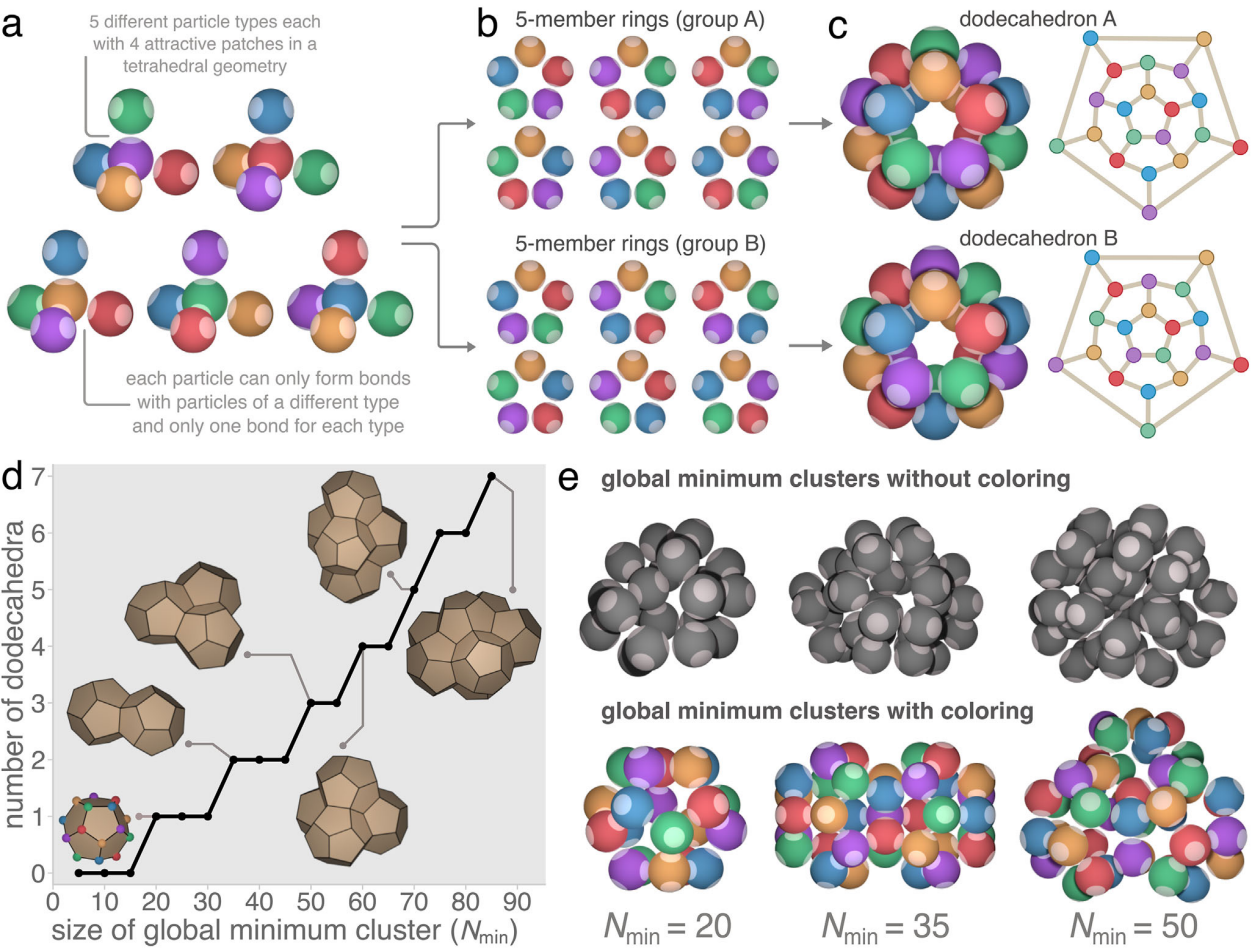
Programming geometric frustration. a) Design of tetrahedral patchy particle system favoring the formation of dodecahedra: five particle types where each particle can form a single bond with a particle of another type and no bonds with particles of the same type. b) The system can form 12 unique five‐member rings, where each ring belongs to one of two groups (A or B) depending on whether they form a face of dodecahedron A or B (shown in panel (c)). A graph representation of each dodecahedron is shown to their right, where each circle represents a particle (and is colored accordingly) and bonded particles are connected by lines. d) Number of dodecahedra in the global minimum clusters of the patchy particle system shown in panel (a) as a function of *N*_min_. The insets show visualizations of select minima where five‐member rings are represented as solid faces. e) Snapshots of global minimum clusters on the potential energy landscape of size *N*_min_ = 20, 35 and 50 for the colored system presented in panel (a) (bottom) and for a system where all interactions are equal (top). Without coloring the clusters contain a mixture of ring sizes while the clusters of the colored system contain only five‐member rings.

Here, through coloring, we design a system of tetrahedral patchy particles – particles with a repulsive core and four attractive patches in a tetrahedral arrangement – which self‐assemble into an amorphous structure possessing dodecahedral medium‐range order. **Figure** **1**a shows our “designer” tetrahedral patchy particles, which locally favor the formation of five‐member rings and dodecahedral cages. The system is a quinary mixture of five distinct particle types (indicated by different colors in Figure 1a), with each type having four uniquely colored patches. The interaction between these patches is such that each particle can form a single bond with a particle of each different type and no bonds with particles of the same type. We note that Liu et al. recently synthesized a four‐component mixture of DNA patchy particles which self‐assembled into a cubic tetrastack crystal, we therefore envision our particles being realized experimentally using similar DNA‐based patchy particles.^[^ ^11^ ^]^

Figure 1b shows the twelve possible five‐member rings which can be formed by the quinary system, where the rings have been separated into two groups of six. The rings in each of the two groups can be combined to produce two different dodecahedral cages, as shown in Figure 1c. Each of the dodecahedra's twelve faces correspond to a five‐member ring in its associated group, and faces on opposite sides of the dodecahedra correspond to the same five‐member ring rotated clockwise by an angle of π/5. Note that each particle in an isolated dodecahedral cage has three bonds, leaving one patch free for further bonding.

**Figure 2:**
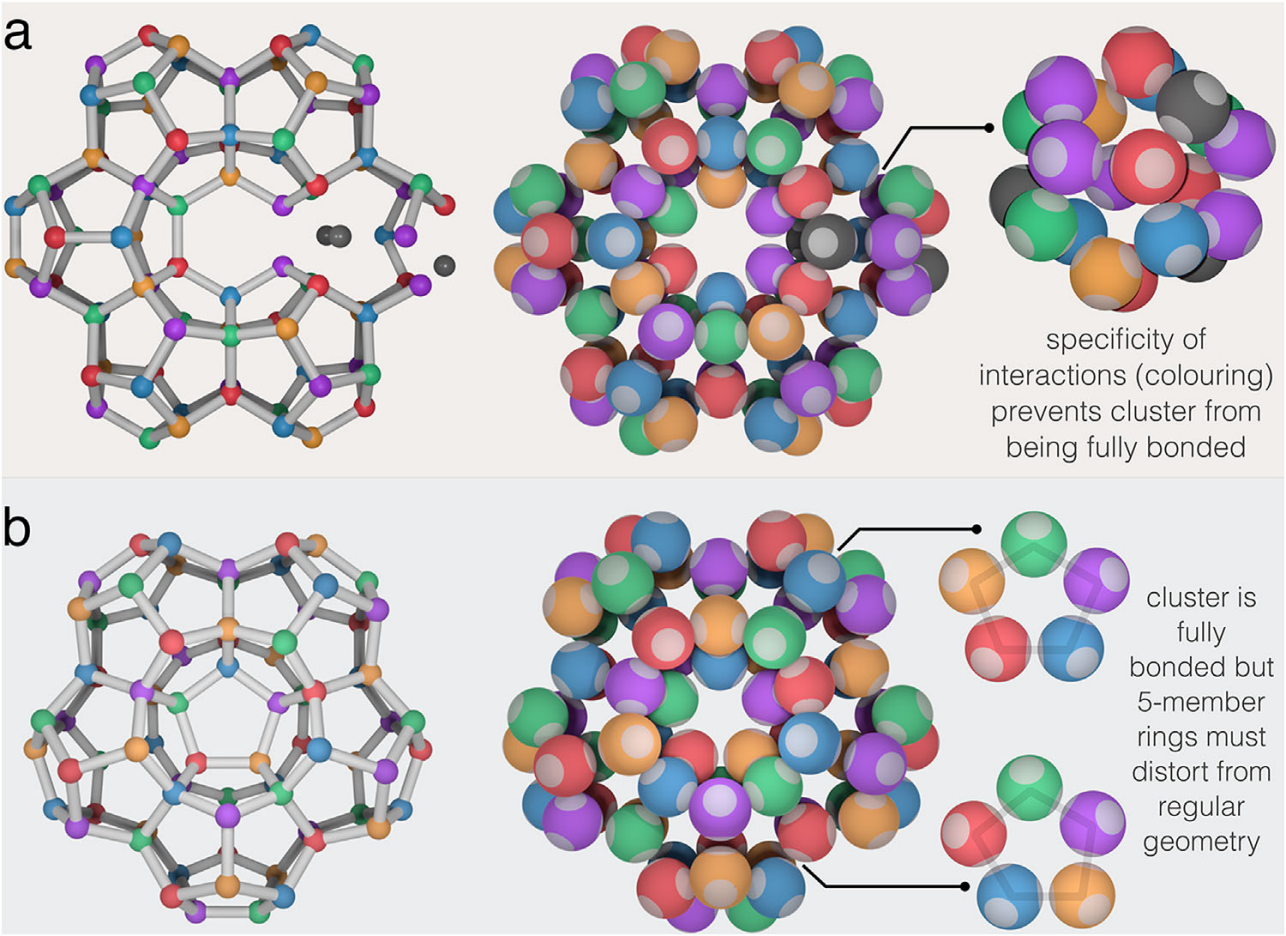
Color frustration. Clusters of dodecahedra forming a loop of (a) six and (b) five face‐sharing dodecahedra. The loop of six face‐sharing dodecahedra is key structural motif of the clathrate s‐II crystal. On the left‐hand side of each panel a network representation of the clusters is shown where the nodes represent particle centers and the cylinders connect particles which are bonded. The loop of six dodecahedra in (a) is color frustrated and so is not fully bonded. The central six‐member ring is incompatible with the coloring of the quinary system. The gray sites cannot be occupied by bond‐forming particles since color‐imposed binding rules cannot be satisfied. The loop of five dodecahedra in (b) is fully bonded, however the five‐member rings comprising the dodecahedra must distort to ensure every bond is formed.

To show that these dodecahedra represent locally favored structures of the quinary system, we use basin‐hopping global optimization^[^ ^65^ ^]^ to identify minimum energy clusters containing an equal number of each particle type on the potential energy landscape. Figure 1d shows the number of dodecahedra in the putative global minima as a function of the cluster size (*N*_min_). All identified global minima are structures constructed from five‐member rings and (once *N*_min_ ⩾ 20) dodecahedra. Figure 1e compares the structure of select global minimum clusters for a one‐component patchy particle system with four identical patches (uncolored) with those of the quinary system. The comparison reveals that clusters with multiple ring sizes – which are the global minima in the absence of coloring – are destabilized in the quinary system due to the specific nature of the interactions leading to unsatisfiable bonds. The coloring thus preferentially selects clusters containing only five‐member rings and dodecahedra as they are maximally bonded. We refer to this suppression of structures incompatible with the bonding rules as color frustration. Color frustration also impacts the stability of crystal structures typically associated with tetrahedral patchy particles (i.e., cubic diamond, hexagonal diamond, clathrate s‐I and clathrate s‐II).^[^ ^23^, ^49^ ^]^ We have verified these crystals cannot be fully bonded in the quinary system due to color frustration. As an example, **Figure** **2**a shows how color frustration impacts the bonding network of a cluster which forms part of the clathrate s‐II structure.^[^ ^49^ ^]^ The cluster is a loop of six face‐sharing dodecahedral cages. Since each cage is rotated by π/5 relative to its neighbors (see Figure 1c), the faces of the first and fifth dodecahedra that would be shared with the sixth are identical. This places particles of the same type next to each other resulting in unsatisfiable bonds and color frustration. Instead, Figure 2b shows a cluster which is a loop of five face‐sharing dodecahedra and so does not display color frustration, but does show the effects of geometric frustration. To ensure maximum bonding the five‐member rings in the cluster must distort from their ideal geometry, leading to an elastic penalty which will frustrate the growth of a space filling structure.

**Figure 3:**
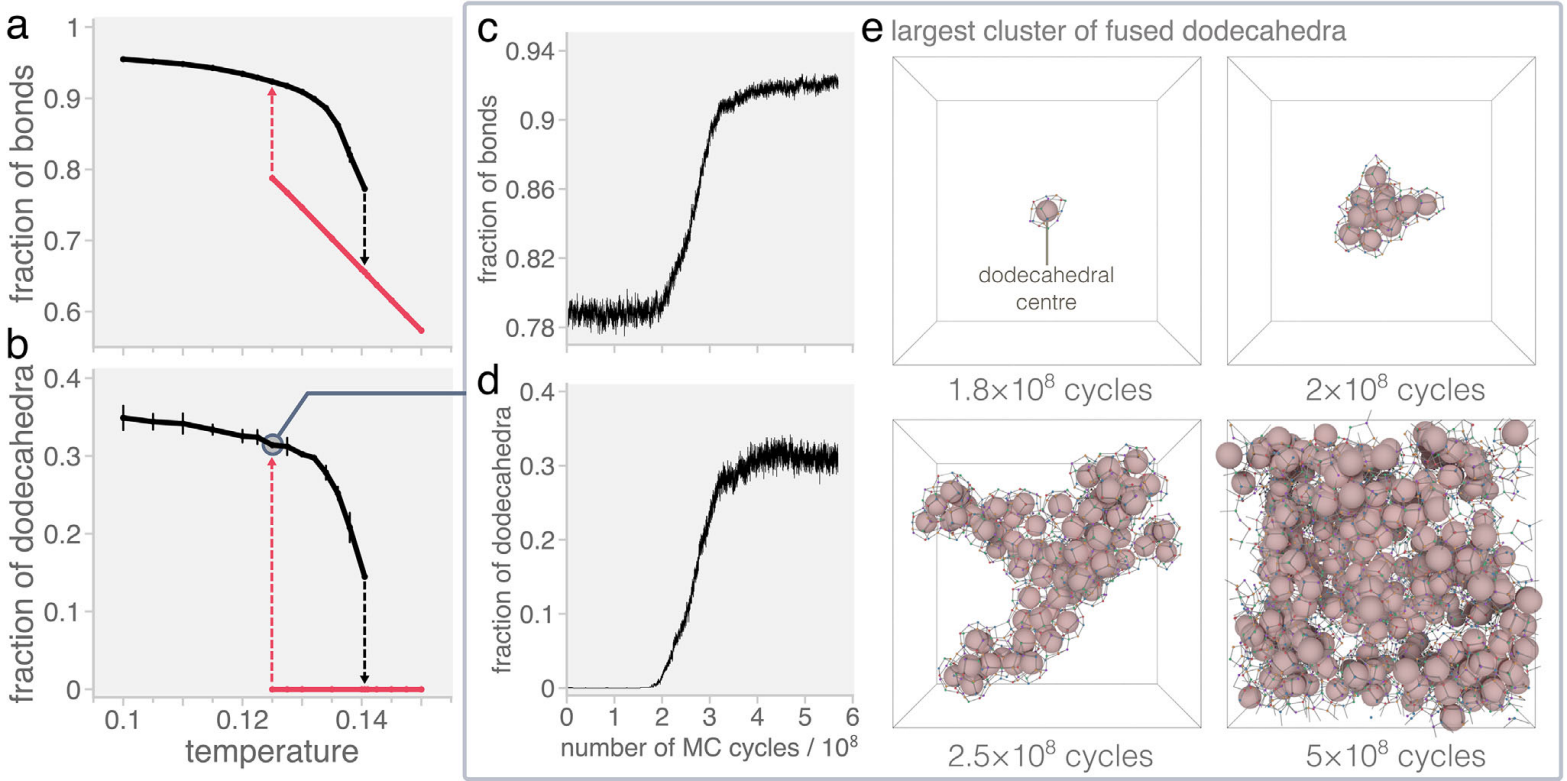
Nucleation of a frustrated phase. a) Fraction of bonds and b) dodecahedra formed in a quinary system of *N* = 5000 patchy particles as a function of temperature (T* = *k*_B_ *T*/ɛ, where *k*_B_ is the Boltzmann constant and ɛ is the well‐depth of the patch‐patch interactions) at a density of ρ* = *N*σ^3^/*V* = 0.4 (where σ is the particle diameter and *V* is the volume of the system). The red upward arrows indicate solidification upon cooling and the black downward arrows indicate melting upon heating. c) Fraction of bonds and d) dodecahedra formed in a quinary system of *N* = 5000 tetrahedral patchy particles along a Monte Carlo trajectory at T* = 0.125 and ρ* = 0.4. e) Representative snapshots of the largest cluster of fused dodecahedra (i.e., dodecahedra which share a face) at different times along the trajectory. The large pinkish spheres represent the centers of the dodecahedral clusters.

As compared to the uncolored system, color frustration changes the energy landscape by destabilizing crystalline configurations and disordered liquid configurations rich in non‐pentagonal rings. What is then left of the landscape? To answer this question, we numerically investigate the self‐assembly behavior by performing Monte Carlo simulations in the canonical (*NVT*) ensemble using a system of *N* = 5000 patchy particles (with an equal number of each particle type) at a number density of ρ* = *N*σ^3^/*V* = 0.4 (where σ is the particle diameter and *V* is the volume of the system) for various temperatures T* = *k*_B_ *T*/ɛ (where *k*_B_ is the Boltzmann constant and ɛ is the well‐depth of the patch‐patch interactions). **Figure** **3**a,b show the temperature dependence of the fraction of possible bonds *f*_b_ = *N*_b_/(2*N*) (where *N*_b_ is the total number of bonds formed) and dodecahedra *f*_dod_ = 20*N*_dod_/(4*N*) = 5*N*_dod_/*N* (where *N*_dod_ is the total number of dodecahedra formed, 20 is the number of particles in a dodecahedron, and *four* is the maximum number of dodecahedra a particle can belong to), respectively. At high temperatures, where entropy plays an important role in selecting ring sizes, the system forms a liquid phase with no dodecahedral cages and rich in six‐ and seven‐ member rings (see Figure S1, Supporting Information).

As we decrease the temperature, color frustration starts to take an effect and below a threshold temperature (T* ≈ 0.13) there is a sudden jump in the number of bonds and dodecahedra, see Figure 3a,b. The system undergoes a first‐order phase transition from a liquid into a phase lower in energy (as signaled by the formation of more bonds) and rich in dodecahedra, which we refer to as the “frustrated phase”. To verify the discontinuous nature of the observed phase transition we conduct simulations in which fully assembled frustrated phases are gradually heated. Figure 3a,b show pronounced hysteresis in both the bond fraction and fraction of dodecahedra, indicating that the system follows different pathways upon heating and cooling. To further probe the melting behavior, Figure S2 (Supporting Information) presents the time evolution of these order parameters along a representative trajectory at T* = 0.1405. Following a slight initial decrease, both quantities remain stable over an extended plateau, demonstrating that the frustrated phase is metastable at this temperature. Eventually, both the bond fraction and the number of dodecahedra drop sharply, marking a first‐order transition into the disordered liquid.

To assess the robustness of our design and the effects of color frustration, we test whether varying the size of the patches has any effect on the formation of the frustrated phase. For all systems considered we observe the spontaneous assembly of a network of face‐sharing dodecahedra, indicating that the formation of the frustrated phase is robust to changes in patch geometry. These findings are detailed in Figure S3 (Supporting Information).

Figure 3c,d show the evolution of the fraction of bonds and dodecahedra, respectively, along a trajectory at T* = 0.125. They show that the frustrated phase appears through a nucleation and growth process. Figure 3e presents snapshots for the largest cluster of fused dodecahedra (i.e., face‐sharing dodecahedra) at select times along the trajectory (see also Video S1, Supporting Information). The snapshots reveal nucleation begins with a single dodecahedral cage that can be of type A or B (see Figure 1c). Once formed, this initial dodecahedron templates the growth of a random network of face‐sharing dodecahedra which – due to color frustration – must all be of the same type. Figure 3e also shows that branches form as the network grows. This branching is due to geometric frustration (i.e., the inability of regular dodecahedra to tile 3D space^[^ ^66^ ^]^) and resembles the stress induced branching of 2D crystals on curved surfaces, which occurs due to elastic stress induced by the Gaussian curvature of the surface.^[^ ^67^, ^68^ ^]^

Geometric frustration is often associated with self‐limiting assembly, where growth is arrested due to accumulating stress.^[^ ^69^, ^70^, ^71^ ^]^ To investigate whether the network of face‐sharing dodecahedra assembled by our system can grow beyond a finite size we perform additional simulations in which a large cluster of the frustrated phase nucleates from a liquid droplet in coexistence with a dilute gas of patchy particles (see Figure S4, Supporting Information). To test for unrestricted growth, we progressively expand the simulation volume and incrementally introduce additional particles into the gas phase, ultimately reaching a system size of *N* = 60000. As shown in Figure S4 (Supporting Information), the network of dodecahedra continues to grow with system size demonstrating that the assembly process is not self‐limiting. This sustained growth confirms that the frustrated phase is not a finite‐sized structure but instead represents a bona‐fide thermodynamic phase formed via a true first‐order phase transition.

**Figure 4:**
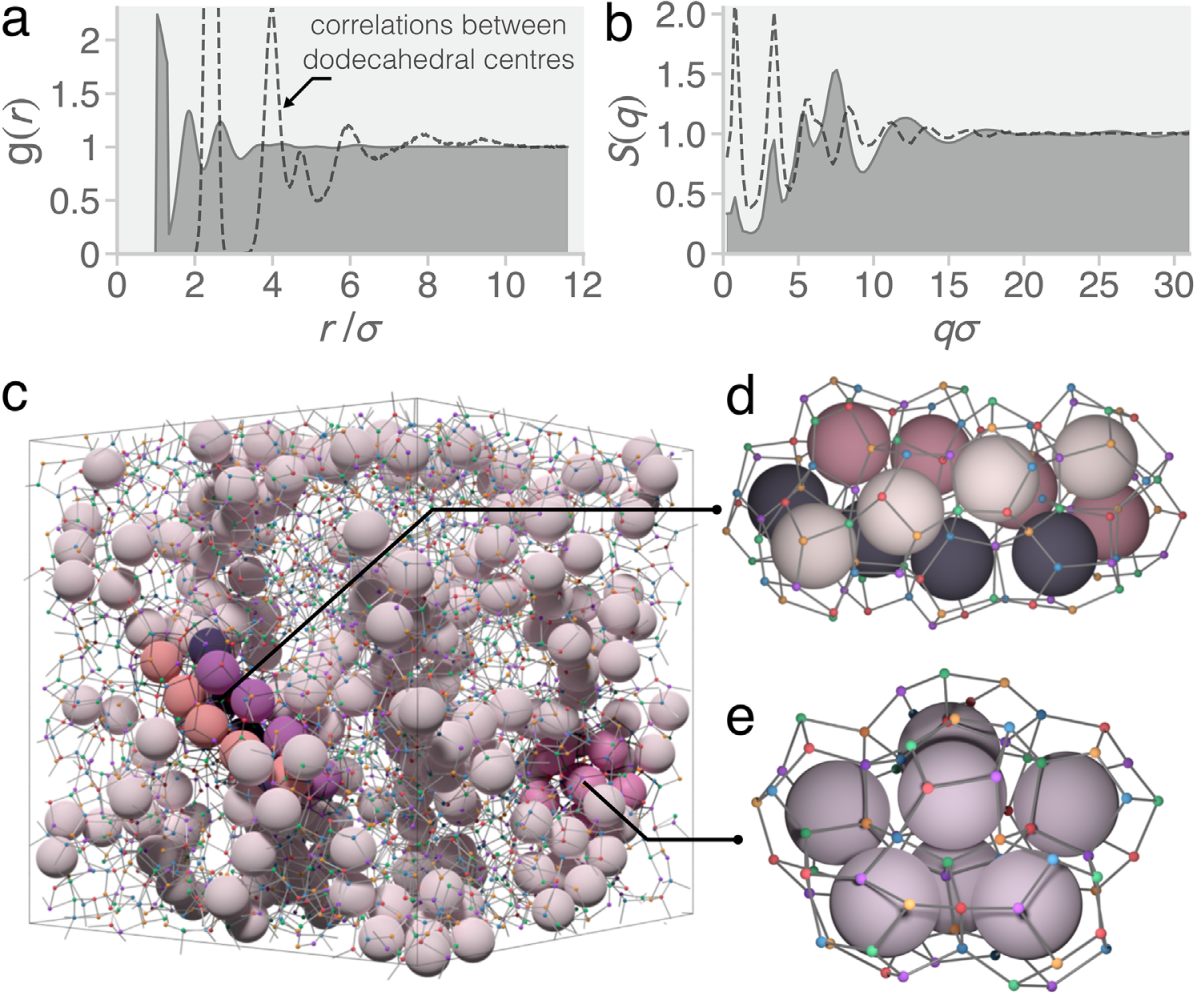
Structure of the frustrated phase. a) Radial distribution function *g*(*r*) and b) static structure factor *S*(*q*) of the metastable liquid phase at T* = 0.1. The solid lines show correlations between the patchy particles while the dashed lines show correlations between the dodecahedral centers. c) Representative snapshot of the frustrated phase at a temperature of T* = 0.1 and a density of ρ* = 0.4 where key structural motifs have been highlighted. Patchy particles are shown as small spheres and bonded particles are connected by gray cylinders. The centers of dodecahedral cages are indicated by the large spheres. d) Bernal spiral of dodecahedral cages. e) Pentagonal bipyramid of dodecahedral cages.

To better understand the structure of the frustrated phase we calculate the particle center radial distribution function *g*(*r*) and static structure factor *S*(*q*), shown in **Figure** **4**a,b, respectively. The radial distribution function suggests that the phase does not possess long‐range order due to the lack of well‐defined peaks at large distances. The static structure factor displays three “pre‐peaks” at *q*σ ≈ 1, 3, and 5 in addition to the primary peak at *q*σ ≈ 7.5. The presence of such pre‐peaks is an indication of medium‐range order and is a hallmark of covalent glasses such as SiO_2_ or GeSe_2_.^[^ ^37^, ^38^, ^72^ ^]^ Here, they signal medium‐range order arising due to the formation of face‐sharing dodecahedra, as shown by the static structure factor calculated using the centers of the dodecahedra in Figure 4b. Figure 4c shows a representative snapshot of the frustrated phase where the network of dodecahedral cages has been highlighted. Figure 4d,e show two key structural motifs associated with the medium‐range order in the network, Bernal spirals of dodecahedra and pentagonal bipyramids of dodecahedra (the latter also shown in Figure 1d for *N*_min_ = 85), respectively. Additional dodecahedra are bonded to these motifs, but they cannot fill the space due to geometric frustration. The space remaining in‐between the branches of the dodecahedral network is filled with a disordered network that includes partially completed dodecahedral cages.

**Figure 5:**
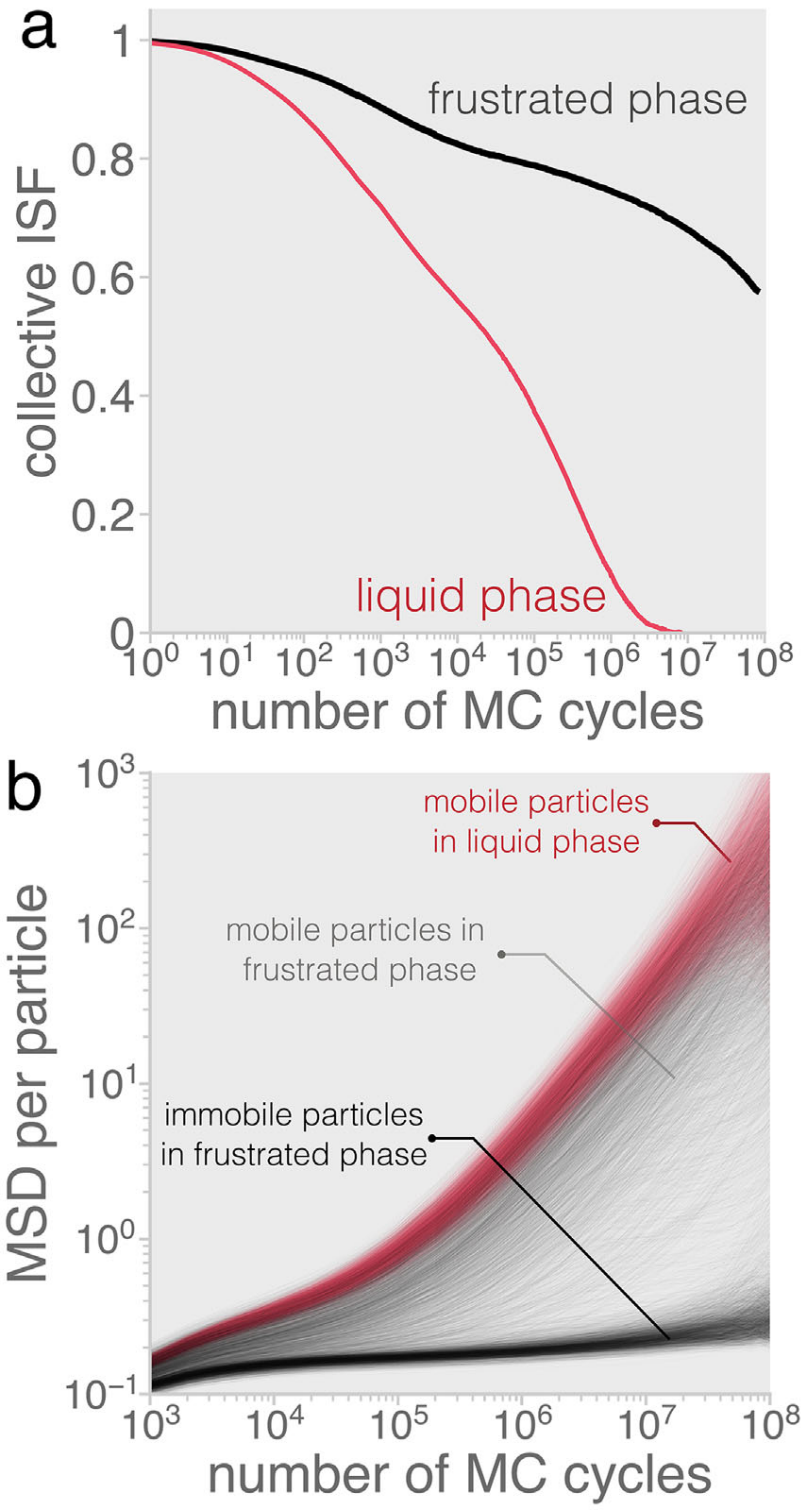
Glassy dynamics in the frustrated phase. Comparing the dynamics of the frustrated phase to the liquid from which it nucleates. a) Collective intermediate scattering function for the liquid phase and frustrated phase at T* = 0.1225, and *q*σ ≈ 3. b) Mean‐squared displacement (MSD) of individual particles in the liquid and frustrated phases. Particles in the frustrated phase form a narrow flat band at small values of the MSD and a disperse band spanning a range of MSD values. Particles in the first band are designated “immobile” and all others are considered “mobile”.

Since a dodecahedron exhibits icosahedral symmetry, the formation of a disordered network of dodecahedra inevitably draws comparison to glass‐forming systems where geometric frustration arises due to the formation of locally preferred structures (e.g., icosahedral clusters) incompatible with the requirements for long‐range periodic order.^[^ ^47^, ^73^ ^]^ In the case of glasses these locally preferred structures are continuously generated upon cooling and do not arise from a first‐order phase transition. The frustration they induce keeps the material in a disordered state, even as it cools and solidifies, resulting in the bypassing of crystallization. We investigate the dynamical behavior of the frustrated phase and compare it to that of the liquid from which it nucleates. **Figure** **5**a shows the collective intermediate scattering function of the two phases. The frustrated phase displays dynamics orders of magnitude slower than the liquid and does not relax on currently accessible timescales. Figure 5b shows the mean squared displacement (MSD) for individual particles in the liquid and frustrated phase. Liquid particles form a single band and display diffusive behavior at long times. In the frustrated phase there is heterogeneous dynamic behavior, a subset of particles display confined motion and another diffusive motion. The “immobile” particles are associated with dodecahedral clusters while the “mobile particles” are not. It is possible for mobile and immobile particles to exchange position, however, this exchange leaves the dodecahedral cage unmodified (see Figure S5, Supporting Information).

## 3 Discussion and Conclusion

In summary, we have designed a system that locally favors the formation of five‐member rings and dodecahedral cages – structures which induce geometric frustration – and encoded color frustration to disfavor crystalline configurations. We discover that this system nucleates into a new frustrated phase from the liquid. Nucleation starts with a single dodecahedral cage which templates the growth of a 3D network of face‐sharing dodecahedra that are dynamically arrested. This is in stark contrast to typical scenarios of geometric frustration where structural motifs that cannot tile Euclidean space emerge continuously upon supercooling,^[^ ^73^, ^74^ ^]^ as originally noted by Frank in the case of icosahedral ordering.^[^ ^75^ ^]^ The frustrated phase does not possess long‐range order due to geometric frustration, however, medium‐range order appears in the form of extended clusters of dodecahedral cages such as pentagonal bipyramids and Bernal spirals.

In contrast to glasses, which are generated through rapid quenching and remain intrinsically out of equilibrium, the frustrated phase is a thermodynamically stable disordered material. This distinction is critical in the context of material design. For glasses the resulting microstructure is highly sensitive to the kinetic pathway, this makes it challenging to engineer specific structural features. By employing programmed self‐assembly, rather than relying on kinetic trapping, our approach offers a path to reliably engineer amorphous materials with tailored structural and functional properties.

We can establish a set of general design principles for programming the self‐assembly of disordered structures. Most importantly, the building block design should locally favor the formation of a structural motif that cannot tile Euclidean space, leading to geometric frustration. This structural motif must also have open boundaries to allow for unrestricted growth and avoid self‐limiting assembly, as can often arise in the presence of geometric frustration.^[^ ^70^ ^]^ Additionally, the design should disfavor (or better yet prohibit) the formation of any crystalline structures that may compete with the disordered structure. In this work, all these requirements have been satisfied via color frustration.

Much like liquid crystals and quasicrystals, the frustrated phase reported here appears to be a new intermediate state of matter. It does not possess long‐range translational order and so it is not crystalline, neither is there orientational order and so it cannot be quasi‐crystalline. The phase is disordered, although it has medium‐range order, and its dynamical behavior is arrested. However, it differs from glasses produced by supercooling a liquid since it forms through a nucleation and growth process. As such, we argue that this is a new class of equilibrium phase underpinned by both color and geometric frustration. Our results open new pathways for utilizing frustration to engineer new amorphous materials and pushes the boundaries of nanotechnology beyond traditional periodic self‐assembly.

## 4 Experimental Section

### Patchy Particle Models

The Kern‐Frenkel pair potential,^[^ ^76^ ^]^ which has been extensively used to model patchy particles,^[^ ^7^, ^21^, ^22^, ^23^, ^24^, ^25^, ^26^, ^28^, ^29^, ^49^ ^]^ was employed to study bulk phase behavior. Since the Kern‐Frenkel potential is discontinuous, to explore the potential energy landscape, a continuous version of the Kern‐Frenkel potential was used.^[^ ^77^ ^]^ The details of these two models are outlined below.

### Kern‐Frenkel Model

Each spherical particle was considered to have hard core, surface was decorated with four circular attractive patches in a tetrahedral symmetry. The effective potential for a pair of patchy particles *u*_*ij*_ is given by:

(1) $$u_{ij}\left(r_{ij},\Omega_{i},\Omega_{j}\right)=u_{\text{hs}}\left(r_{ij}\right)+u_{\text{sw}}\left(r_{ij}\right)\sum_{\alpha ,\beta =1}^{4}f\left(r_{ij},{\hat{n}}_{i,\alpha },{\hat{n}}_{j,\beta }\right)$$

where *r*_*ij*_ = |**r**_*ij*_| is the center‐to‐center distance between particles *i* and *j* and $\Omega_{i}$ and $\Omega_{j}$ describe the orientations of particles *i* and *j*, respectively. ${\hat{n}}_{i,\alpha }$ is a normalized vector from the center of particle *i* in the direction of the center of patch α on its surface, and thus depends on $\Omega_{i}$. Similarly, ${\hat{n}}_{j,\beta }$ is a normalized vector from the center of particle *j* in the direction of the center of patch β, and thus depends on $\Omega_{j}$. *u*_hs_ is the hard‐sphere pair potential

(2) $$u_{\text{hs}}\left(r_{ij}\right)=\left\{\begin{array}{cc} \infty & \;\text{if}\;r_{ij}<\sigma \\ 0 & \;\text{otherwise} \end{array}\right.$$

with σ representing the hard‐sphere diameter. *u*_sw_ is a square‐well potential given by

(3) $$u_{\text{sw}}\left(r_{ij}\right)=\left\{\begin{array}{cc} -\varepsilon & \;\text{if}\;\sigma \leq r_{ij}\leq \left(1+\delta \right)\sigma \\ 0 & \;\text{otherwise} \end{array}\right.$$

where ɛ is the well‐depth of the patch–patch interactions and δ controls the range of the attraction. Finally, the factor $f(r_{ij},{\hat{n}}_{i,\alpha },{\hat{n}}_{j,\beta })$ controls the angular dependence of the interaction between two patches on distinct particles, and is given by

(4) $$f\left(r_{ij},{\hat{n}}_{i,\alpha },{\hat{n}}_{j,\beta }\right)=\left\{\begin{array}{cc} 1 & \text{if}\;{\hat{n}}_{i,\alpha }\cdot {\hat{r}}_{ij}>\cos\theta \;\text{and}\;{\hat{n}}_{j,\beta }\cdot {\hat{r}}_{ji}>\cos\theta \\ 0 & \text{otherwise} \end{array}\right.$$

The width of the patches was controlled by θ, which represents the half‐opening angle of the patches. Here, δ = 0.3 and cos θ = 0.86 unless otherwise stated (as is the case in Figure S3, Supporting Information). Reduced units were used such that length was in units of σ, the energy in units of ɛ, the temperature in units of ɛ/*k*_B_, with the Boltzmann constant *k*_B_ taken to be equal to one.

### Continuous Patchy Particle Model

For the global optimization calculations a continuous patchy particle model was employed,^[^ ^77^ ^]^

(5) $$\begin{array}{cc} u_{ij}\left(r_{ij},\Omega_{i},\Omega_{j}\right) & =u_{y}\left(r_{ij},{\hat{u}}_{i},{\hat{u}}_{j}\right)+u_{p}\left(r_{ij}\right)\sum_{\alpha ,\beta =1}^{4}\frac{\left(1+\Phi_{\alpha }\right)\left(1+\Phi_{\beta }\right)}{4} \end{array}$$

The repulsive core–core interaction is given by the repulsive Yukawa potential,

(6) $$\begin{array}{cc} u_{y}\left(r_{ij}\right) & =\varepsilon_{y}\frac{\exp[-\kappa \left(r_{ij}-\sigma \right)]}{r_{ij}/\sigma } \end{array}$$

where κ is the inverse Debye screening length, ɛ_y_ is the Yukawa contact potential and σ is the diameter of the particles. The attractive interaction between the patches has two components controlling distance and angular dependence. The distance dependence of the potential is given by,

(7) $$\begin{array}{cc} u_{p}\left(r_{ij}\right) & =\left\{\begin{array}{cc} -\varepsilon & \text{if}\;\left(r_{ij}-\lambda \right)<0\\ -\frac{1}{2}\varepsilon \left[1+\cos\left(\pi \left(r_{ij}-\lambda \right)s\right)\right] & \text{if}\;0\leq \left(r_{ij}-\lambda \right)\leq s^{-1}\\ 0 & \text{otherwise} \end{array}\right. \end{array}$$

where λ is the largest separation at which the patch–patch attraction is strongest and the parameter *s* controls the range over which the patches interact. The angular dependence is given by,

(8) $$\begin{array}{cc} \Phi_{\alpha } & =\left\{\begin{array}{cc} -1 & \text{if}\;-{\hat{r}}_{ij}\cdot {\hat{n}}_{i,\alpha }<\cos\phi \\ -\cos\left(\frac{\pi [-{\hat{r}}_{ij}\cdot {\hat{n}}_{i,\alpha }-\cos\phi ]}{1-\cos\phi }\right) & \text{otherwise} \end{array}\right. \end{array}$$

and

(9) $$\begin{array}{cc} \Phi_{\beta } & =\left\{\begin{array}{cc} -1 & \text{if}\;{\hat{r}}_{ij}\cdot {\hat{n}}_{j,\beta }<\cos\phi \\ -\cos\left(\frac{\pi [{\hat{r}}_{ij}\cdot {\hat{n}}_{j,\beta }-\cos\phi ]}{1-\cos\phi }\right) & \text{otherwise} \end{array}\right. \end{array}$$

where ${\hat{n}}_{i,\alpha }$ is a normalized vector from the center of particle *i* in the direction of the center of patch α on its surface, ${\hat{n}}_{j,\beta }$ is a normalized vector from the center of particle *j* in the direction of the center of patch β on its surface and the parameter cos ϕ controls the range of angles over which the patches interact (i.e., it effectively controls the width of a patches).

Here, ɛ_*y*_ = ɛ = 1, κ = 50, λ = 1.02, *s* = 1, and ϕ = π/4 were used. Again, reduced units were used such that length was in units of σ and energy in units of ɛ_*y*_.

### Global Optimization Calculations

A series of global optimization calculations were performed on the potential energy landscape using basin‐hopping global optimization^[^ ^65^ ^]^ for clusters of particles of sizes *N*_min_ ∈ [5 − 85] interacting through the continuous patchy particle model described above. Energy minimization at each basin‐hopping step was performed with the L‐BFGS algorithm,^[^ ^78^, ^79^ ^]^ using analytical derivatives. The rotational coordinates of the particles were parameterized using quaternions, with the corresponding derivatives taken with respect to angle‐axis vectors using the exponential map of quaternions.^[^ ^80^ ^]^ For cluster sizes *N*_min_ ⩽ 20, a single global optimization run of up to ten million basin‐hopping steps were carried out, for cluster sizes 20 < *N*_min_ ⩽ 60, ten independent global optimization runs of 2.5 million basin‐hopping steps and for larger clusters were carried out, twenty independent global optimization runs of three million basin‐hopping steps were carried out.

### Monte Carlo Simulations

All Monte Carlo simulations were carried out with systems of patchy particles (interacting via the Kern‐Frenkel potential) contained in a cubic box under periodic boundary conditions, using the minimum image convention, in the canonical (*NVT*) ensemble. Monte Carlo simulations were performed with two possible moves, roto‐translations and aggregation‐volume‐bias (AVB).^[^ ^81^ ^]^ Roto‐translations simply involve the rotation and translation of a random particle in a random direction. AVB moves involved displacing a random particle into the vicinity of another so that a bond can form between them. To ensure ergodicity was not broken the inverse move could also be performed where a random bond between two particles was broken. Each patchy particle was treated as a rigid body whose orientational degrees of freedom were represented by a rotation matrix. Additionally, to ensure that each particle only forms one bond per patch, a repulsive three‐body term was introduced to the system's Hamiltonian.^[^ ^28^, ^82^ ^]^ The potential energy was calculated using spherical cutoff of 1.3σ and a cell‐list was used for efficiency. The simulations start with particles randomly positioned in the box such that there were no overlaps and with random orientations.

### Ring Statistics

To identify rings in a system of patchy particles, the adjacency matrix *A*_*ij*_ was first extracted for each configuration, where two particles *i* and *j* were considered connected by an edge if they shared a patch–patch interaction. Once the adjacency matrix of the system was computed, shortest‐path rings were identified. Starting from each node *i* of the patchy particle network, the shortest path connecting any two of its bonded neighbors *l* and *m* (which did not include *i*) was extracted, then adding node *i* to this path produces a shortest‐path ring. This was then repeated for all pairs of bonded neighbors *l* and *m*. If there were multiple “shortest” paths of the same length connecting *l* and *m*, then all resulting rings were added to the set of shortest‐path rings.

### Identification of Dodecahedral Cages

To identify dodecahedral cages in the bonding network of patchy particles we followed the approach outlined by Jacobson et al.^[^ ^83^ ^]^ The process begins by identifying all five‐member (pentagonal) rings. For each ring whether it could serve as the base of a “cup” was determining by assessing if it shares an edge with five other pentagonal rings. A cup is a configuration of six pentagonal rings – encompassing a total of 15 particles – which could be thought of as a dodecahedral cage with one pentagonal ring missing. A list of all possible cups was then generated. Each pair of cups was subsequently compared to determine whether they could be combined to form a dodecahedral cage. Successful combinations occur when the ten particles along the rims of the two cups are identical and arranged in the same order. To ensure uniqueness, duplicate dodecahedra – arising from different choices of the 12 potential base rings – were eliminated, resulting in a final list of all distinct dodecahedra.

## Conflict of Interest

The authors declare no conflict of interest.

## Supporting information

Supporting Information

Supporting Video S1

## Data Availability

The data that support the findings of this study are available from the corresponding author upon reasonable request.
