# Supplementary material for: Designing the Self‐Assembly of Disordered Materials Via Color Frustration
Source: Adv Mater. 2025 Jun 10;37(34):2502136. doi: 10.1002/adma.202502136 (PMC12392867; doi:10.1002/adma.202502136)
Supplement: Supplementary file 1 — Supporting Information [file ADMA-37-2502136-s001.pdf]

ADVANCED MATERIALS

Supporting Information

for *Adv. Mater.*, DOI 10.1002/adma.202502136

Designing the Self-Assembly of Disordered Materials Via Color Frustration

Andreas Neophytou, Francesco Sciortino and John Russo*

Designing the self-assembly of disordered materials via color frustration

Andreas Neophytou[1], Francesco Sciortino[1], and John Russo[1]

[1]Dipartimento di Fisica, Sapienza Università di Roma, Piazzale Aldo Moro 5, 00185 Roma, Italy

Supporting Figures

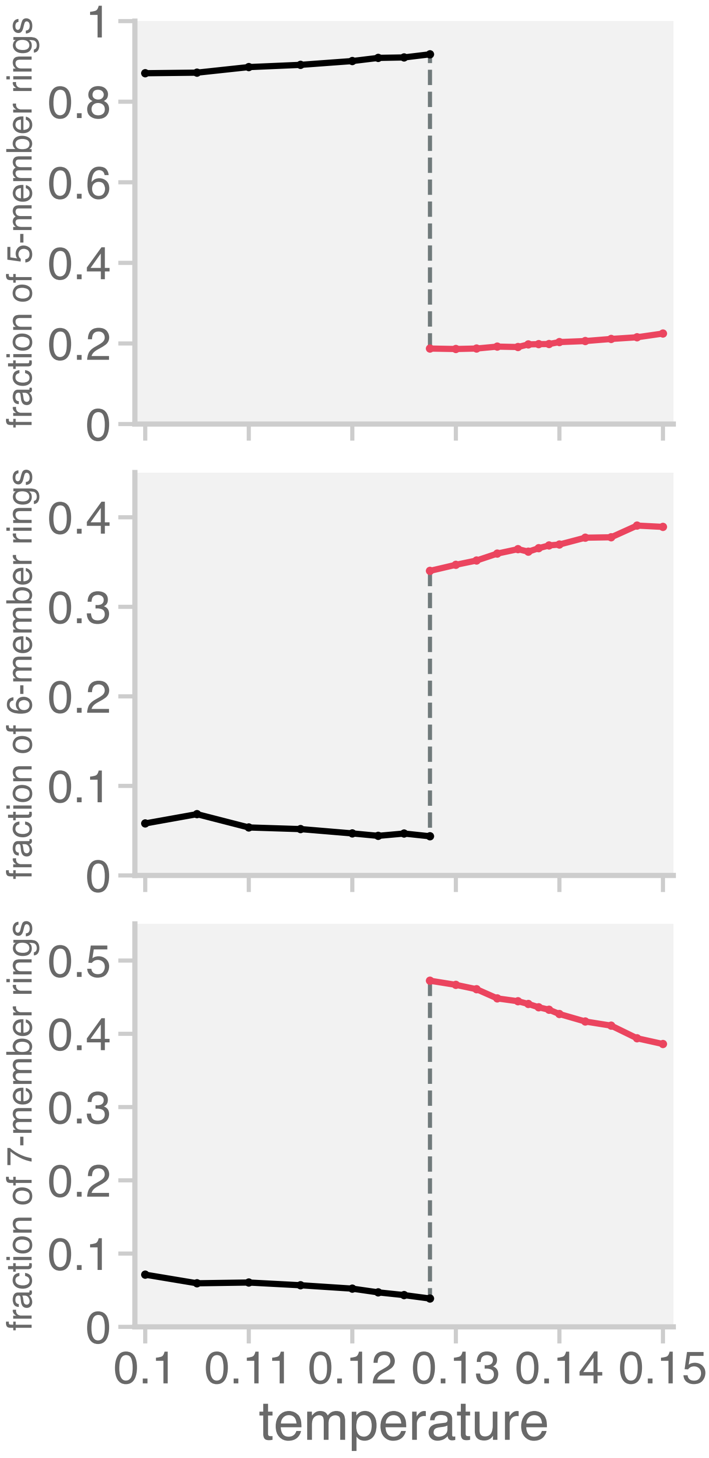

Figure S1: **Ring statistics for the quinary system of patchy particles.** The fraction of rings in a quinary system of $N = 5000$ patchy particles as a function of temperature at a density of $\rho^* = 0.4$ identified to be (**top**) 5-, (**middle**) 6-, and (**bottom**) 7-member rings.

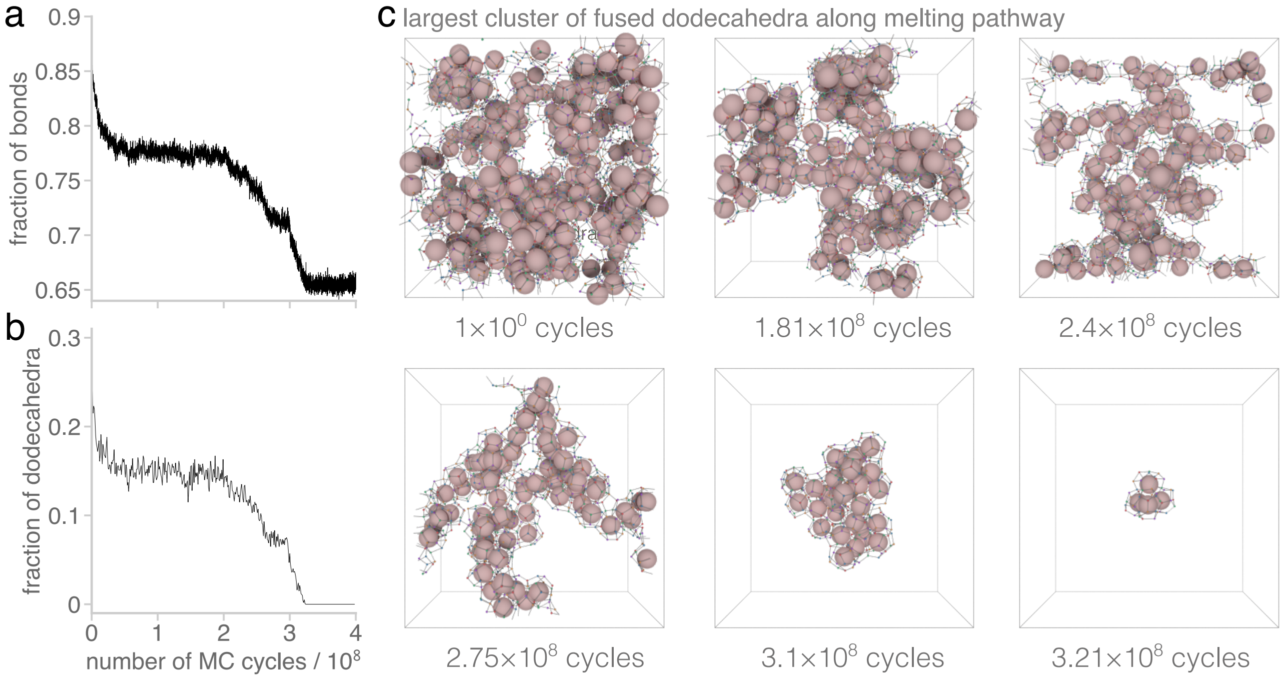

Figure S2: **Melting of the frustrated phase.** (**a**) Fraction of bonds and (**b**) dodecahedra present in a quinary system of $N = 5000$ tetrahedral patchy particles along a Monte Carlo trajectory at $T^* = 0.1405$ and $\rho^* = 0.4$. The system begins in the frustrated phase which was self-assembled at $T^* = 0.13$, it is then heated to a temperature of $T^* = 0.1405$. (**c**) Representative snapshots of the largest cluster of fused dodecahedra (*i.e.*, dodecahedra which share a face) at different times along the trajectory. The large pinkish spheres represent the centers of the dodecahedral clusters.

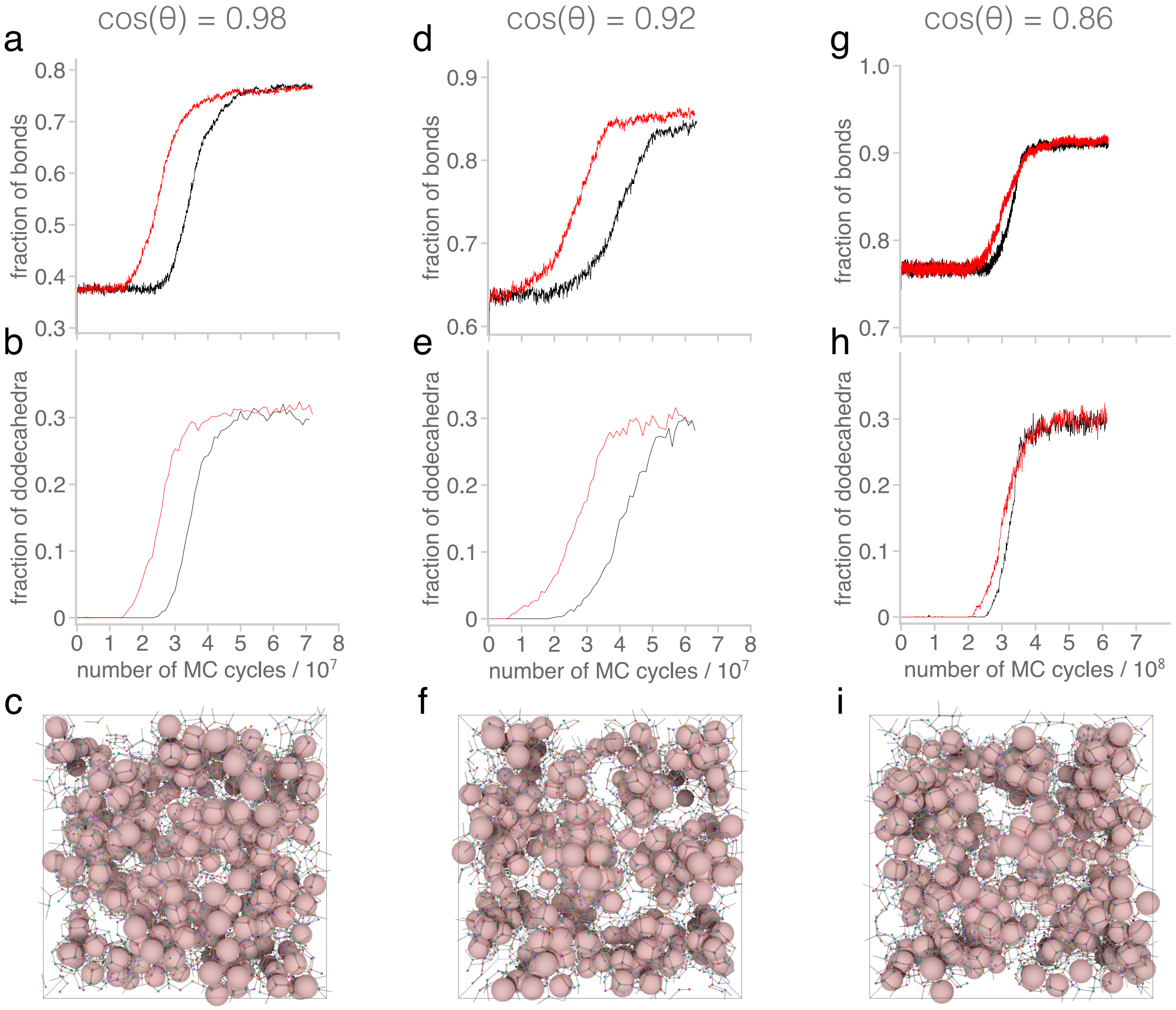

Figure S3: **Robustness of color frustration.** Self-assembly of the frustrated phase for quinary systems of $N = 5000$ patchy particles at a density of $\rho^* = 0.4$ with $\delta = 0.3$ and (**a-c**) $\cos\theta = 0.98$ at $T^* = 0.105$, (**d-f**) $\cos\theta = 0.92$ at $T^* = 0.125$ and (**g-i**) $\cos\theta = 0.86$ at $T^* = 0.1275$. Panels **a**, **d** and **g** show the evolution of the fraction of bonds $f_b = N_b/(2N)$ (where N_b is the total number of bonds) formed for two representative trajectories for each system. Panels **b**, **e** and **h** show the evolution of the fraction of dodecahedra $f_{dod} = 5N_{dod}/N$ (where N_{dod} is the total number of dodecahedra) formed for two representative trajectories for each system. Panels **c**, **f** and **i** show representative snapshots of the frustrated phase for each system where the dodecahedra centers are visualised as large pink spheres.

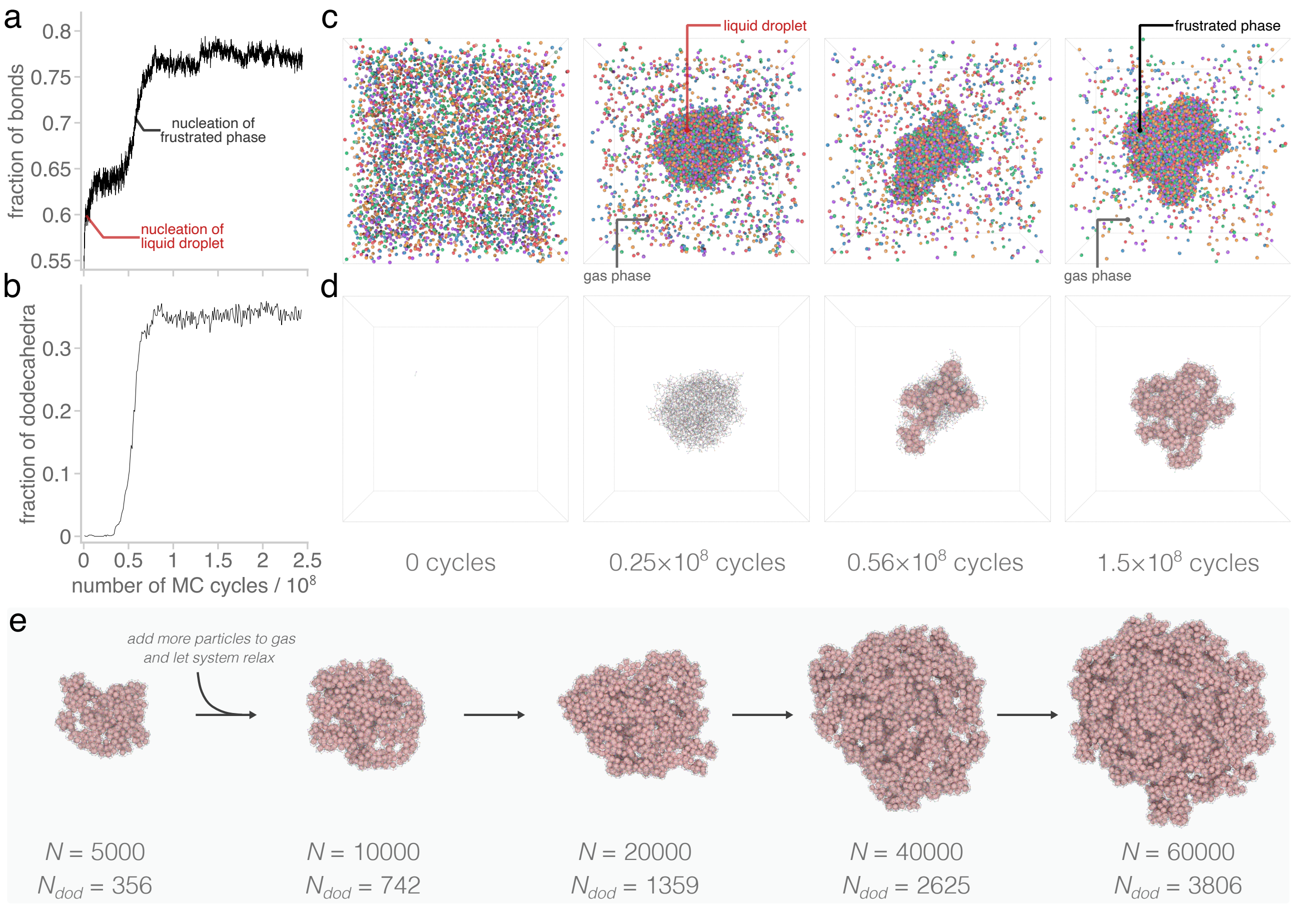

Figure S4: **Unrestricted growth of the frustrated phase.** (**a**) Fraction of bonds and (**b**) dodecahedra along a Monte Carlo trajectory for a system of $N = 5000$ particles at a density of $\rho^* = 0.02$ and temperature of $T^* = 0.115$. Snapshots showing the (**c**) patchy particles and (**d**) largest bonded cluster in the system at select times along the trajectory. The large pink spheres represent dodecahedral centers. (**e**) Snapshots of large clusters of the frustrated phase for different system sizes ranging from $N = 5000$ to 60000 patchy particles. The clusters are grown by adding additional particles to gas phase of the system shown in panels **a-d**, these larger systems are then allowed to relax resulting in a growth of the number of dodecahedra (N_{dod}) in the cluster.

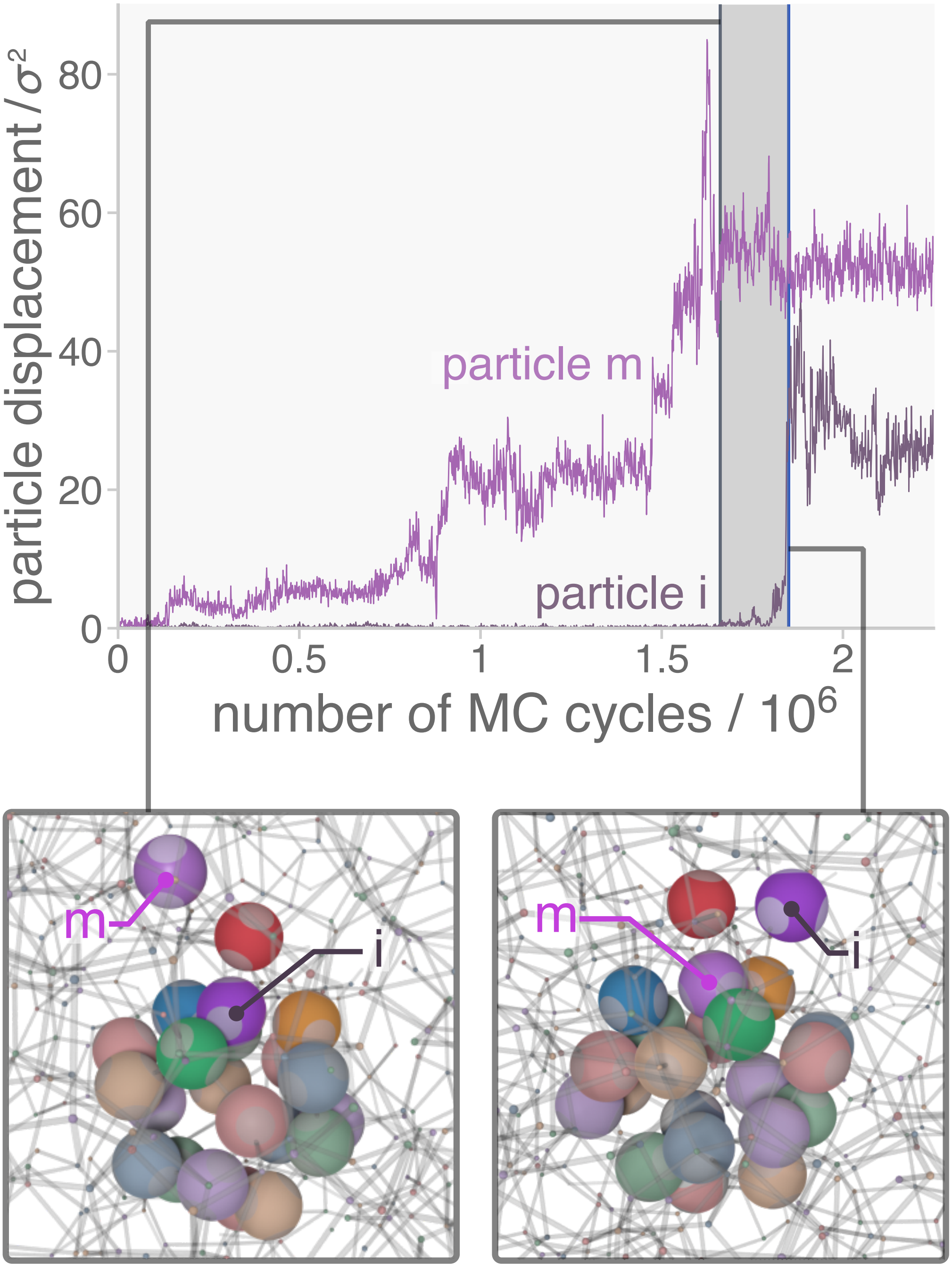

Figure S5: **Mobile-immobile particle swap.** Squared displacement of two particles, labelled i and m, along a trajectory. The highlighted region signifies the time window over which particle m (which is initially mobile) replaces particle i (which is initially immobile) in the dodecahedral network. Snapshots of the system, focusing on particles i and m and the associated dodecahedral cage, at the beginning and end of swapping window. Note that over this time window the identity of all other highlighted particles does not change and the centre-of-mass of the dodecahedral cage does not change.

Supporting Videos

Supporting Video 1 Largest cluster of fused dodecahedral cages (where the dodecahedra are represented as large spheres) along a Monte Carlo trajectory at a temperature of $T^* = 0.125$ and a density of $\rho^* = 0.4$.
